# Synthetic Food dyes cause testicular damage via up-regulation of pro-inflammatory cytokines and down-regulation of FSH-R and TESK-1 gene expression

**DOI:** 10.5935/1518-0557.20200097

**Published:** 2021

**Authors:** Iheanyichukwu Wopara, Emmanuel U Modo, Samuel Kelechi Mobisson, G Adebayo Olusegun, EB Umoren, Blessing O Orji, Philippe E Mounmbegna, Stephanie Okoye Ujunwa

**Affiliations:** 1 Department of Biochemistry, Faculty of Basic Medical Sciences, PAMO University of Medical Sciences, Nigeria; 2 Department of Biochemistry, Faculty of Sciences, Madonna University, Nigeria; 3 Department of Human Physiology, Faculty of Basic Medical Sciences, Madonna University, Nigeria; 4 Department of Physiology, Faculty of Basic Medical Sciences, PAMO University of Medical Sciences, Nigeria; 5 Department of Biochemistry and Molecular Biology, Federal university Dutsin-ma, Katsina State, Nigeria

**Keywords:** Orchitis, Tartrazine, Erythrosine, Testicular Genes and Luteinizing hormone

## Abstract

**Objective:**

This study investigated the effects of Tartrazine and Erythrosine (T+E) on the reproductive hormones and expression of some pro-inflammatory cytokines and testicular genes in testis of male Wistar rats.

**Methods:**

25 male Wistar rats (150-180g) were divided into 5 groups (n=5). Group 1 received distilled water while groups 2, 3, 4 and 5 were treated with T+E (2.5mg/kg, 5mg/kg, 10mg/kg and 20mg/kg) for the period of 23 days. Toxicity studies of the combined dye were investigated by evaluating serum reproductive hormones [Follicle stimulating hormone (FSH), Luteinizing hormone (LH), Testosterone], gene expression and profiling, and testes histology.

**Results:**

male Wistar rats (150-180g) were divided into 5 groups (n=5). Group 1 received distilled water while groups 2, 3, 4 and 5 were treated with T+E (2.5mg/kg, 5mg/kg, 10mg/kg and 20mg/kg) for the period of 23 days. Toxicity studies of the combined dye were investigated by evaluating serum reproductive hormones [Follicle stimulating hormone (FSH), Luteinizing hormone (LH), Testosterone], gene expression and profiling, and testes histology.

**Conclusions:**

This present study reveals that the dyes could impair testicular function as evident in the up-regulation of pro-inflammatory cytokines and down-regulation of TESK-1 gene expression and architecture of the testes leading to Orchitis.

## INTRODUCTION

Food dyes have been extensively used as food additives, especially in beverages and candies. There are various natural colors; however, their tint is dependent on pH and the color can be lost during time and storage conditions (Amchova *et al*., 2015). A wide range of food coloring or additives, running into more than 2,500 items used to preserve or enhance food is a consequence of industrialization and the development of food processing technology (Toledo, 1996; Hirschbruch & Torres 1998). They come in many forms consisting of liquids, powders, gels, and pastes. Food coloring is used both in commercial food production and in domestic cooking. They are also used in a variety of non-food applications, including cosmetics, pharmaceuticals, home craft projects, and medical devices (Delwiche, 2004). A food additive is only approved for human consumption after studying its acute, sub-acute and chronic toxicity. Several metabolites of these substances, such as nitrous compounds, have been found to be carcinogenic (Cemek *et al.*, 2014). Toxicity or the benefits of these colorants depend on to what extent the food components affect absorption, excretion or the metabolism as a whole (Cemek *et al.*, 2014). Because there may be interaction among different substances of food colorants, the definition of adequate safety limits for human consumption is further compounded (Preussmann, 1978; Ferguson, 1999). Among the various synthetic food colorants, Tartrazine and Erythrosine have been selected for their it possible toxicity effect on the testes.

Tartrazine is a synthetic lemon yellow azo dye primarily used as a food-coloring agent (Elhkim *et al*., 2007). It is also known as E number E102, C.I. 19140, FD&C Yellow 5, Acid Yellow 23, Food Yellow 4, and trisodium 1-(4-sulfonatophenyl)-4-(4-sulfonatophenylazo)-5-pyrazolone-3-carboxylate). Products containing tartrazine commonly include processed commercial foods that have an artificial yellow or green color, or that consumers expect to be brown or creamy looking. It has been frequently used in the bright yellow coloring mimicking “lemon” color in baked goods. Types of pharmaceutical products that may contain tartrazine include vitamins, antacids, cold medications (including cough drops and throat lozenges), lotions and prescription drugs.

Erythrosine, also known as Red No. 3, is an organ iodine compound, specifically a derivative of fluorone. It is cherry-pink synthetic, primarily used for food coloring (Lyday & Kaiho, 2015). It is a disodium salt of 2, 4, 5, 7-tetraiodofluorescein and its maximum absorbance is at 530 nm in an aqueous solution, and it is also subject to photo degradation (Gurr, 1971). Erythrosine (E127) is commonly used in sweets, such as candies and popsicles, and even more widely used in cake-decorating gels. These synthetic food dyes have been implicated with high serum or tissue concentration which could lead to diabetes, increase tumor cell growth, reproductive toxicity, increase blood glucose, decrease percentage of high density lipoprotein cholesterol (HDL-C), decrease plasma immune-system agents, increase plasma lipid lipoprotein and total cholesterol, increase oxidative stress, etc. (Dixit & Goyal, 2013; Dafallah *et al*., 2015; Merinas-Amo *et al.,* 2019). In this study, the effects of these synthetic food colorants were evaluated to assess the possible changes on the expression of pro-inflammatory genes and the testicular gene functioning in male rats.

## MATERIALS AND METHODS

### Laboratory animals

We used twenty (25) male albino Wistar rats aged 8 weeks and weighing 150-180g for this study. The animals were housed in the Department of biochemistry Animal house, Madonna University, Nigeria. Standard animal cages with wood dust as bedding were used to keep the animals. They were allowed *ad libitum* access to rat chow and clean water, and exposed to 12/12-hr light/dark cycle. The animals were acclimatized for 7 days. The animals were kept in line according to the principles for animal care, as prescribed in the Helsinki’s 1964 Declaration, and as stipulated in the National Academy of Science (NAS) “Guide for the Care and Use of Laboratory Animals” published by the National Institute of Health.

### Reagent Preparation

We got the Tartrazine (T0388-100G) and Erythrosine (1159360025) samples from Sigma Company, USA; 2.5mg/kg body weight, 5mg/kg b.wt, 10mg/kg b.wt, 20mg/kg b.wt, of each sample were weighed and dissolved in distilled water.

### Experimental design and prepared colorant administration

The rats were randomly assigned according to their weight into five groups (n=5) of five each. The first group served as control; the second, third, fourth and fifth groups were administered with 2.5mg/kg, 5mg/kg, 10mg/kg and 20mg/kg body weight of a combined mixture of Tartrazine and Erythrosine respectively. The dyes were administered via orogastric feeding, once a day for 23 days, while the control group received distilled water, after which the animals were slaughtered under chloroform anesthesia and their testis were harvested and put in an Eppendorf tube containing triazol, to assess some pro-inflammatory and testicular gene expression following a method recently used by Wopara *et al*. (2019).

### Assessment of Serum Reproductive Hormones

The serum obtained from the blood samples was analyzed to determine the concentrations of FSH, LH, and testosterone. Serum testosterone, luteinizing hormone and follicle stimulating hormone concentrations were determined using the ELISA kit for rats, following the method described by the kit manufacturer (Immunometrics Limited UK), as used by Mobisson *et al*. (2018).

Principle: The ELISA method is based on the principle of high specificity of antibodies to bind molecules, which in this case are the different reproductive hormones. The antibody is tagged with an enzyme, since the enzyme-labelled antibody reacts with the hormone. The concentration of hormone present in the sample is obtained by introducing a substrate for the enzyme, which forms a colored product. The intensity of the color seen is proportional to the concentration of the bound hormone.

### Gene expression and profiling assessment

#### 2.4.1 Total RNA Isolation

Total RNA was isolated from the whole tissue following a method recently used by Omotuyi *et al*. (2018) and Wopara *et al.* (2020).

##### Procedure

The tissues were homogenized in cold (4˚C) TRI reagent and the total RNA was partitioned in Chloroform, and the mixture centrifuged at 15,000 rpm/15min. Then, the RNA from the clear supernatant was precipitated using an equal volume of isopropanol and RNA pellets were rinsed twice in 70% ethanol (70ml absolute ethanol). The pellets were air dried for five mins and dissolved in buffer (1Mm sodium citrate, pH 6.4).

#### cDNA Conversion

##### Procedure

Total RNA quantity (concentration ( = 40* ) and quality ( 1.8) was assessed using the ratio of / (A=absorbance), read using spectrophotometer. The DNA contamination was removed from RNA following DNAse 1 treatment (NEB, Cat; M0253S), as specified by the manufacturer. 2µl solution containing 100mg of DNA-free RNA was converted to cDNA using M-MuLV Reverse transcriptase kit (NEB, Cat: M0253S) in 20 final volume (2 µl, random primer mix; 2µl, 10X M-MuLV buffer; 1µl, M-MuLV RT (200 U/ µl); 2µl, 10mMdNTP; 0.2µl, RNAse Inhibitor (40U/µl) and 10.8µl nuclease-free water). The reaction proceeded at room temperature O/N. Inactivation of M-MulV Reverse transcriptase was performed at 65˚C/20min.

#### PCR amplification and agarose gel electrophoresis

PCR amplification for the determination of genes whose primers (Primer3 software) are listed below was done using the following protocol:

PCR amplification was performed in a 25µl volume reaction mixture containing 2µl cDNA (40mg), 2µl primer (100pmol) 12.5µl Ready Mix Taq PCR master mix (One Taq Quick-load 2x, master mix, NEB, Cat: M0486S) and 8.5µl nuclease-free water. Initial denaturation at 95˚C for 5 minutes was followed by 20 cycles of amplification (denaturation at 95˚C for 30 seconds and annealing (see TM values for each primer pair on table 1) for 30 seconds and extension at 72˚C for 60 seconds) and ending with final extension at 72˚C for 10 minutes. In all experiments, negative controls were included where the reaction mixture had no cDNA. The amplicons were resolved on 2.0% agarose gel (Cleaver Scientific Limited: Lot: 14170811) in Tris (RGT reagent, china, Lot: 20170605)-Borate (JDH chemicals, China, Lot 20141117)-EDTA buffer (pH 8.4).

**Table 1:** List of Primers

Parameters	Forward primer	Reverse primer
**IL-1beta**	TTGAGTCTGCACAGTTCCCC	TCCTGGGGAAGGCATTAGGA
**IL-1a**	CTT CAC ATC CGC AGC TTT CC	GCG AGT GAC TTA GGA CGA GG
**TNF-a**	CACTGGCTGTGTCATTGCTC	TCTGCCAGTTCCACAT CTCG
**FSH-R**	ACCAGGAAGTGCCATAACCC	CAAACCTCAGTTCAATGGCG
**LH-R**	CTC CGT GGA CTC CCA AAC AA	CAG GGT GAT AAC CGT CAG GG
**TESK-1**	ACCGTTTACATCGTGGCTGG	CCCTGTGAATGGCGTTTGTC
**CYCLOPHILIN**	TGGAGAGCACCAAGACAGACA	TGCCGGAGTCGACAATGAT

#### Amplicon image processing and semi-quantification

The in-gel amplicon band images captured on camera were processed on a Keynote platform. Gel density quantification was done using the Image-J software. Each point represent relative expression ((test gene band intensity/internal control band intensity)* 100) plotted using the Numbers software (Mac OSX version).

##### Histological Studies

The testes from the control and from the rats which received combined doses of colorants were removed carefully, cleared of connective tissues and fixed in Boiun’s fluid [0.2% picric acid/2% (v/v) formaldehyde in PBS] for 48 hours. The sections were obtained and stained with hematoxylin and eosin (H & E) stains. The microscopic slides were labeled appropriately. Photomicrographs were taken at x125 and ×500 magnifications using a light microscope (Leica DM 750, Switzerland).

##### Statistical Analysis

All the data is presented as Mean+Standard Error of Mean (SEM). The statistical analysis was done using one-way analysis of variance (ANOVA) and then followed by the Newman-Keuls post hoc test. All statistical analyses were done using the Prism software, version (Diego, CA, USA). The statistical difference at a *p* level <0.05, <0.01, and <0.001were considered significant.

## RESULTS

### The effects of a combined Tartrazine and Erythrosine mixture on the serum concentration of follicle stimulating hormone after 23 days of treatment

**Figure 1:**
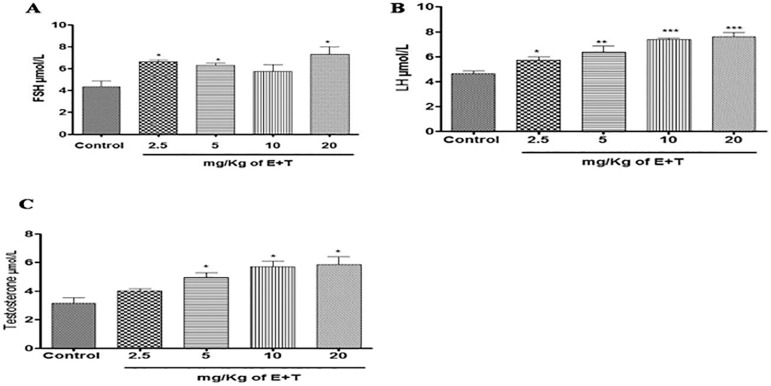
(A, B & C). Effects of the combined mixture of T+E on serum concentration of FSH, LH & Testosterone in rats. The values are expressed as mean ± SEM, n=4. The level of significance was expressed as *p<0.05, **p<0.01, and ***p<0.001 compared with the control group. T+E = Tartrazine and Erythrosine

Repeated treatment with Tartrazine and Erythrosine (T+E) produces an increase in the serum FSH concentration in rats. There was a significantly (*p*<0.05) increased serum FSH concentration in the rat treated with 2.5mg/kg T+E, 5mg/kg T+E and 20mg/kg T+E compared to the control. However, the group treated with 10mg/kg T+E increased but there was no significant difference when compared to the control animals (Figure 1A).

### The effects of a combined Tartrazine and Erythrosine mixture on serum concentration of luteinizing hormone after 23 days of treatment

The result obtained from repeated treatment with Tartrazine and Erythrosine after 23 days showed improved serum concentration of LH in the rats. There was a significant [(*p*<0.05) and (*p*<0.01)] increase in the group treated with 2.5mg/kg T+E and 5mg/kg T+E compared with the untreated group. Moreover, the groups treated with 10mg/kg T+E and 20mg/kg T+E also had a significantly (*p*<0.001) increased serum concentration of LH when compared to the control group after 23 days of repeated T+E treatment (Figure 1B).

### The effects of a combined mixture of Tartrazine and Erythrosine on serum concentration of Testosterone after 23 days of treatment

From these results, there was a significant (*p*<0.05) increase in the serum concentration of Testosterone in the rats treated with 5mg/kg T+E, 10mg/kg T+E and 20mg/kg T+E when compared against the control group. Furthermore, the group treated with 2.5mg/kg T+E showed no significant effect when compared to the control group (Figure 1C).

### The effects of a combined mixture of Tartrazine and Erythrosine in the expression of follicle stimulating hormone gene after 23 days of treatment

**Figure 2:**
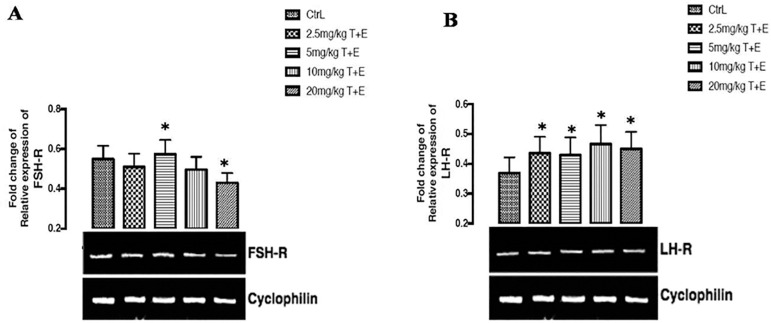
(A & B). Effects of a combined mixture of T+E in the expression of FSH & LH receptor gene in rats. The values are expressed as mean ± SEM, n=4. The level of significance was expressed as *p<0.05, compared with the control group. T+E = Tartrazine and Erythrosine

As shown in Figure 2A below, there was a significant (*p*<0.05) up-regulation in the group treated with 5mg/kg T+E and a significant (*p*<0.05) down-regulation in the group treated with 20mg/kg T+E when compared to the control group. However, there was no significant fold change of relative expression of FSH receptor in the groups treated with 2.5mg/kg T+E and 10g/kg T+E after 23 days of repeated treatment.

### The effects of a combined treatment of Tartrazine and Erythrosine in the expression of luteinizing hormone receptor gene, after twenty days of treatment

Figure 2B below shows the effects of T+E on the fold change of relative expression of LH receptor in the rats after 23 days of repeated treatment. The result shows that there was a significant (*p*<0.05) up-regulation across all the groups treated (2.5mg/kg, 5mg/kg, 10mg/kg, and 20mg/kg) with T+E when compared to the control group.

### The effects of a combined treatment of Tartrazine and Erythrosine on the expression tumor necrotic factor receptor gene, after 23 days of treatment

**Figure 3:**
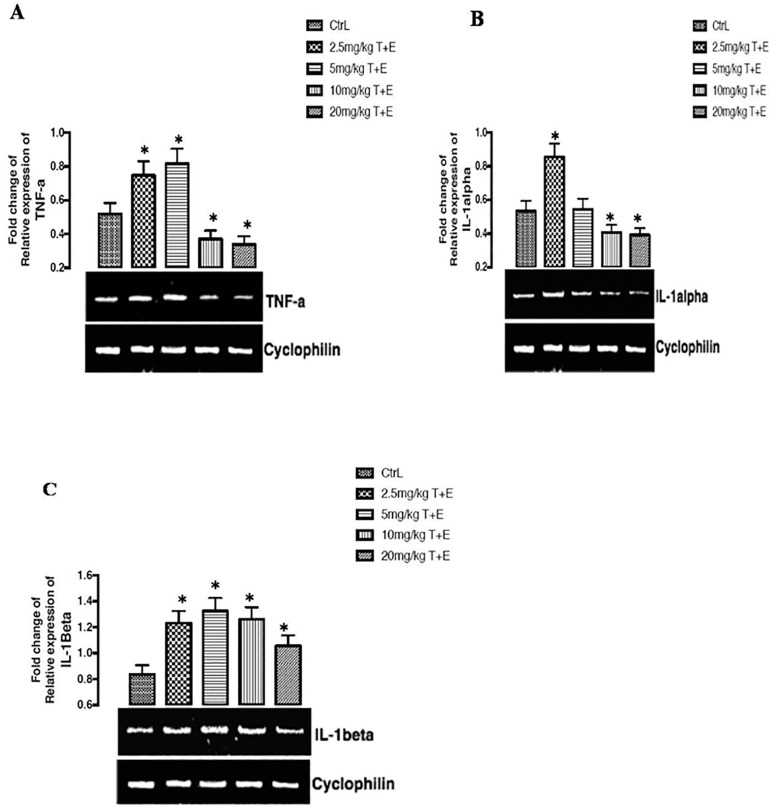
(A, B & C). Effects of a combined mixture of T+E in the expression of TNF-a, IL-1alpha, and IL- 1beta receptor gene in rats. The values are expressed as mean ± SEM, n=4. The level of significance was expressed as *p<0.05, compared with the control group. T+E = Tartrazine and Erythrosine

As shown in figure 3A below, there was a significant (*p*<0.05) up-regulation effect on the expression of tumor necrotic factor genes in the group treated with 2.5mg/kg T+E and 5mg/kg T+E while the groups treated with 10mg/kg T+E and 20mg/kg T+E significantly (*p*<0.05) down-regulated the expression of tumor necrotic factor gene when compared to controls and other groups.

### Effects of a combined mixture of Tartrazine and Erythrosine in the expression of the interlukin1-α gene after 23 days of treatment

The results found in this study reveals that there was both up-regulation and down-regulation on the expression of the interlukin1-α gene after 23 days of treatment in the rats. There was a significant (*p*<0.05) up-regulation of the interlukin1-α gene expression in the group treated with 2.5mg/kg T+E when compared to the control groups. However, a significant (*p*<0.05) down-regulation of the interlukin1-α gene expression was noticed in the groups treated with 10mg/kg T+E and 20mg/kg T+E of when compared to the control group (figure 3B).

### Effects of a combined mixture of Tartrazine and Erythrosine on the expression of the interlukin1-β gene after 23 days of treatment

The combined treatment on the expression of interlukin1-β gene after 23 days of treatment was found to significantly (*p*<0.05) up-regulate the group treated with 2.5mg/kg T+E, 5mg/kg T+E, 10mg/kg T+E and 20mg/kg T+E compared against the control group (figure 3C).

### The effects of a combined treatment of Tartrazine and Erythrosine on the expression of testicular kinase 1 gene after twenty-three days of treatment

**Figure 4:**
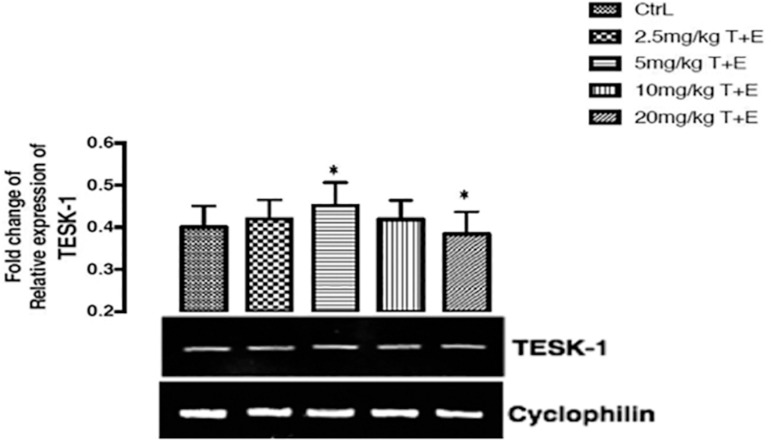
Effects of a combined mixture of T+E in the expression of TESK-1 receptor gene in rats. The values are expressed as mean ± SEM, n=4. The level of significance was expressed as *p<0.05, compared with the control group. T+E = Tartrazine and Erythrosine

The testicular kinase 1 gene expression was significantly (*p*<0.05) up-regulated in the group treated with 5mg/kg T+E and there was a slight significant (*p*<0.05) down-regulation effect shown in the group treated with 20mg/kg T+E when compared to the control group (figure 4).

### Histological Examination of the Testes

**Figure 5:**
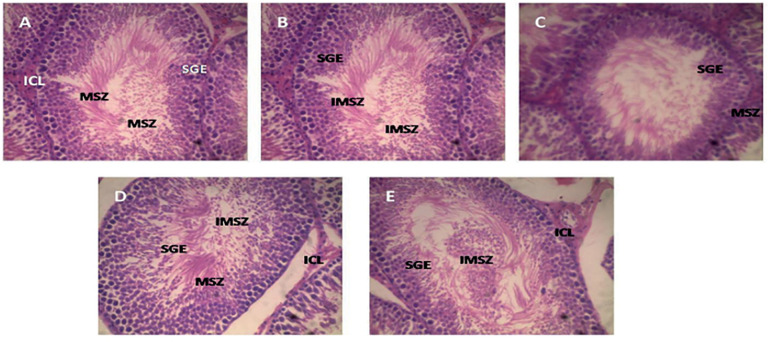
Photomicrograph of the testes in rats treated with the combined mixture of T+E. A=Control group, B= 2.5mg/kg T+E, C=5mg/kg T+E, 10mg/kg T+E, and 20mg/kg T+E. The testes were stained with H&E (presented at X500 magnification). IMSZ=Immature spermatozoa, ICL=Interstitial cells of Leydig, SGE=Stratified germinal epithelium, MSZ=Mature spermatozoa

The architectural examination of the testes in the control group shows that the seminiferous tubules, lined by a stratified germinal epithelium, appear normal, with mature spermatozoa and immature spermatozoa lying freely in the lumen. The interstitial Leydig cells also appear normal (figure 5). In the group treated with 2.5mg/kg T+E and 5mg/kg T+E, the germinal epithelium of the seminiferous tubules reduced in thickness with no visible distortion of the general tissue and its lumen, revealing more of matured and less immature spermatozoa, while the group treated with 10mg/kg T+E only showed variable thickness of the germinal epithelium lining the seminiferous tubules but have all other structural characteristics as the 2.5mg/kg T+E and 5mg/kg T+E, when compared to the control group (figure 5). However, structural examination of the group treated with 20mg/kg T+E reveals flattened and degenerating interstitial Leydig cells with reduced thickness of the germinal epithelium lining the seminiferous tubules and a large number of immature spermatozoa in the lumen of the seminiferous tubules when compared with the control group (figure 5).

## DISCUSSION

This study investigated the toxic effects and biochemical changes in some constituents in serum and testes of experimental rats treated with combined tartrazine and erythrosine that are commonly used in the field of additives in industrial packaged foods or foods largely consumed at home. Tartrazine and erythrosine were previously reported to induce several clinical derangements when consumed moderately or in excess, or even at various recommended dose.

Repeated treatment with combined tartrazine and erythrosine after 23 days showed increased serum reproductive hormones. FSH and LH are gonadotrophic hormones, which are released by the pituitary gland into the bloodstream. They are both essential for puberty development, acting on the Sertoli and Leydig cells in the male testes to stimulate sperm production. Testosterone is the main steroid sex hormone in male Wistar rats; it is secreted by Leydig cells in the testes under complex neuroendocrine interactions and control (Olaniyan *et al*., 2018). Increased secretion of testosterone in the testes is critically required for normal spermatogenesis, development, and maintenance of sperm morphology and normal physiology of the seminiferous tubules. This study revealed that the combined tartrazine and erythrosine administration significantly improved serum FSH, LH and Testosterone, as seen in the rats treated with 2.5mg/kg T+E, 5mg/kg T+E, 10mg/kg T+E and 20mg/kg T+E. Our findings agree with the reports that the administration of synthetic food dyes such as tartrazine at a high dose in rats did not induce deleterious effects on reproductive hormonal parameters, suggesting that with a long-term consumption there was no significant difference, and could possibly enhance production and release of reproductive hormone into the circulation (Tanaka, 2006; Elekima & Nwachuku, 2019). This suggested a possible effect of this combined additives stemming from its testicular androgen biosynthesis capacity.

Cyclophilins are housekeeping genes; they are also called constitutive genes, which are needed for the maintenance of essential cellular functions, and are present in cells both in normal and pathological conditions. Cyclophilin proteins have peptidyl propyl isomerase activity, which catalyzes the peptide bond isomerization in *trans-cis* form of proline residues and facilitates protein folding and trafficking. Recent studies have revealed that Cyclophilin can be secreted by the cells in response to inflammatory stimuli in several human diseases (Nigro *et al*., 2013). Reproductive hormone receptors and pro-inflammatory cytokines were examined in this study using Cyclophilin housekeeping gene expression. The major function of follicle stimulating hormone receptors and Luteinizing hormone receptors is binding to follicle stimulating hormone and luteinizing hormone, which is a key hormone in fertility, because it is implicated with spermatogenesis in males (Asatiani et *al*., 1997). FSH-R gene expression was seen to be up regulated and down regulated in 5mg/kg T+E and 20mg/kg T+E treated groups, while LH-R gene expresses up-regulation in all the treated groups. The up-regulatory effects seen in the LH-R gene may initiate binding with LH, which can stimulate cell proliferation, cell differentiation and steroid hormone production. Furthermore, recent studies suggest that LH-R may stimulate the MAPK pathway, and its activation may be responsible to the modulation of LH-induced steroidogenesis (Angelova *et al*., 2003). However, attenuation of the (FSH-R and LH-R)-mediated signaling stem from the combination of receptor uncoupling and down-regulation (Angelova *et al*., 2003; Jiang *et al*., 2014). Reports from this study point out the combined effect of tartrazine and erythrosine on the up-regulation of both FSH-R and LH-R after 23 days of treatment in rats. The results obtained from pro-inflammatory cytokine receptors (TNF-α, IL-1α and IL-1β) expresses both up-regulation and down-regulation. Stressful condition of the testicular cells promotes inflammation leading to impairments such as orchitis. In testicular-inflammation, cytokines such as TNF-α, IL-1α and IL-1β are produced in significant levels, and are particularly known to aggravate testicular disease progression, and subsequent death. Increased levels of these TNF-α, IL-1α and IL-1β precipitate testicular cell degeneration, leading to reproductive hormone impairment or injury (Suescun *et al*., 2003; Fijak *et al*., 2015). In this study, the administration of combined tartrazine and erythrosine in rats significantly up-regulated the gene expression for the testes level of TNF-α, IL-1α and IL-1β, signifying testicular inflammatory activity in the rats. Treatment with combined tartrazine and erythrosine significantly and dose-dependently down regulated the gene expression on testes treated with TNF-α, and IL-1α in some of the rats (10mg/kg T+E and 20mg/kg T+E), compared to the control group. Our findings corroborate reports on the up-regulatory effects of pro-inflammatory cytokine gene expressions due to testicular infections or damage (Di Paolo & Shayakhmetov, 2016). Excessive production of inflammatory cytokines contribute to inflammatory diseases such atherosclerosis, cancer, renal dysfunction and neurological disease (Scarpioni *et al*., 2016; Bent et *al*., 2018).

Testicular protein kinase 1 is an enzyme that is encoded by the TESK1 gene in humans. The dual specificity protein kinase activity catalyzes auto phosphorylation and phosphorylation of exogenous substrates on both serine/threonine and tyrosine residues. TESK1 contains unique structural features composed of the N-terminal unusual protein kinase domain and C-terminal proline-rich region. TESK1 gene plays a central role at and after the meiotic phase of spermatogenesis (Shinkai et *al*., 2002). We observed up-regulation in testicular protein kinase 1 gene expression in rats treated with 5mg/kg T+E. Moreover, the rats treated with 20mg/kg T+E showed a slight down-regulation in the expression of TESK-1. The down-regulation of the TESK1 may be responsible for the testicular damage effect seen in the high-dose treated rats as shown in the testes architecture.

After 23 days of combined tartrazine and erythrosine administration, histological examination of the testes appeared normal in the rats with 2.5mg/kg T+E, 5mg/kg T+E and 10mg/kg T+E. There were not visible distortions in the testes after staining with hematoxylin and eosin dye. The seminiferous tubules lined by a stratified germinal epithelium appears well preserved with mature spermatozoa and immature spermatozoa lying freely in its lumen. The rats treated with 20mg/kg T+E appeared not well preserved with noticeable degeneration of interstitial cells of Leydig in photomicrograph. This may likely be attributed to a down-regulation of the TESK1 and FSH-R genes and up-regulation of IL-1β genes. Furthermore, a large number of immature spermatozoa seen in the lumen of seminiferous tubules and the degeneration of the interstitial cells of Leydig may confirm the testicular damage in this group. The damage to testicular tissue (especially interstitial cells of Leydig) may suggest a detrimental effect of these food colorants on the testes. Our findings agree with previous reports on the combined or singly effect of tartrazine and erythrosine administration on the testes (Elekima & Nwachuku, 2019).

## CONCLUSION

Despite the desirable and amazing impact made in food, cosmetics, medicines, etc., by tartrazine and erythrosine, their buildup in the body can affect the testis by disrupting the normal state of its functional genes as well as tampering with the fertility hormone receptor genes, which may impair spermatogenesis, hence leading to infertility. More so, their buildup may initiate inflammatory diseases of the testis (orchitis).
